# Regional myocardial perfusion imaging in predicting vessel-related outcome: interplay between the perfusion results and angiographic findings

**DOI:** 10.1007/s00259-022-05948-w

**Published:** 2022-09-02

**Authors:** Teresa Mannarino, Adriana D’Antonio, Roberta Assante, Emilia Zampella, Valeria Gaudieri, Pietro Buongiorno, Valeria Cantoni, Roberta Green, Carmela Nappi, Emanuele Criscuolo, Roberto Bologna, Mario Petretta, Piotr Slomka, Alberto Cuocolo, Wanda Acampa

**Affiliations:** 1 Department of Advanced Biomedical Sciences, University of Naples Federico II, Via Pansini 5, 80131 Naples, Italy; 2 IRCCS Synlab-SDN, Naples, Italy; 3 Department of Medicine, Cedars-Sinai Medical Center, Los Angeles, CA USA; 4 David Geffen School of Medicine, UCLA, Los Angeles, CA USA

**Keywords:** Regional MPI, Outcome, SPECT

## Abstract

**Background:**

Despite myocardial perfusion imaging (MPI) by cadmium-zinc-telluride (CZT) single-photon emission computed tomography (SPECT) camera is largely used in the diagnosis and risk stratification of patients with suspected or known coronary artery disease (CAD), no data are available on the prognostic value of a regional MPI evaluation. We evaluated the prognostic value of regional MPI by the CZT camera in predicting clinical outcomes at the vessel level in patients with available angiographic data.

**Methods and results:**

Five hundred and forty-one subjects with suspected or known CAD referred to 99mTc-sestamibi gated CZT-SPECT cardiac imaging and with available angiographic data were studied. Both regional total perfusion deficit (TPD) and ischemic TPD (ITPD) were calculated separately for each vascular territory (left anterior descending, left circumflex, and right coronary artery). The outcome end points were cardiac death, target vessel-related myocardial infarction, or late coronary revascularization. The prevalence of CAD ≥ 50%, regional stress TPD, and regional ITPD was significantly higher in vessels with events as compared to those without (both *P* < 0.001). The receiver operating characteristics area under the curve for regional ITPD for the identification of vessel-related events was 0.81 (95% confidence interval 0.75–0.86). An ITPD value of 2.0% provided the best trade-off for identifying the vessel-related event. At multivariable analysis, both CAD ≥ 50% and ITPD ≥ 2.0% resulted in independent predictors of events.

**Conclusions:**

Regional myocardial perfusion assessed by the CZT camera demonstrated good reliability in predicting vessel-related events in patients with suspected or known CAD.

## Introduction

Myocardial perfusion imaging (MPI) by cadmium-zinc-telluride (CZT) camera has been largely validated in the diagnosis and risk stratification of patients with suspected or known coronary artery disease (CAD) [1–5]. Noninvasive assessment of stress-induced ischemia, along with the severity of symptoms and presence of significant epicardial coronary stenosis on angiography, are powerful tools in both risk estimation and patient management [6]. The prognostic value of CZT cameras has been demonstrated to be similar as compared to conventional cameras [7, 8]. Patients with normal scans have a trend to the low prevalence of hard cardiovascular events at midterm follow-up, leading to the possibility of a prognosis even better than that of conventional stress single-photon emission computed tomography (SPECT) [9]. Moreover, the greater extension of myocardial hypoperfusion by CZT cameras, the higher risk of major cardiac events, and all-cause mortality [10, 11] have been reported. Some authors [12, 13] have investigated the role of global ischemic total perfusion deficit (ITPD) assessed by traditional SPECT cameras. As well, different studies have analyzed the power of semiquantitative global and regional assessment by SPECT and positron emission tomography/computed tomography (PET/CT) in the identification of obstructive CAD [14–16]. To our knowledge, no data are available on the prognostic value of regional myocardial perfusion evaluated by CZT cameras. Therefore, the aim of our study was to evaluate the prognostic value of regional myocardial perfusion assessed by CZT in predicting clinical outcomes at the vessel level in patients with available angiographic data.

## Material and methods

### Study population

A total of 2206 consecutive patients with suspected or known CAD, were referred to stress CZT-SPECT cardiac imaging between February 2016 and June 2020. For the present investigation, patients (*n* = 132) with previous coronary bypass surgery were excluded. Of the remaining 2074 patients, 620 had available coronary angiography. Patients (*n* = 30) referred to early revascularization (within 3 months) after MPI were not included, leaving 590 patients for the final analysis. Demographic information, as well as clinical history, cardiovascular risk factors, such as diabetes, hypertension, dyslipidemia, smoking history, family history of CAD, chest pain symptoms, electrocardiographic abnormalities, previous history of myocardial infarction or coronary revascularization, were collected. Patients were considered diabetic in the case of a previous diagnosis of diabetes mellitus or treatment with oral hypoglycemic drugs or insulin. Hypertension was defined as a known history of systolic blood pressure > 140 mm Hg or treatment with antihypertensive medication. Dyslipidemia was defined as a known history of dyslipidemia or the use of cholesterol-lowering medication. A positive family history of CAD was defined as the presence of CAD in first-degree relatives. All the data were verified and complemented with demographic and clinical information collected from medical records. The review committee of our institution approved this study, and all patients gave informed consent (“Comitato Etico, Università Federico II”, protocol number 110/17).

### MPI acquisition

All patients underwent gated stress/rest MPI after intravenous injection of 99mTc-sestamibi (185 MBq for stress and 370 MBq for rest images) using a cardio-dedicated SPECT camera (D-SPECT, Spectrum Dynamics, Caesarea, Israel). Stress testing was performed using physical exercise by treadmill or dipyridamole (0.142 mg kg^−1^ × minute^−1^ intravenous over 4 min). All patients were instructed to withhold beta-blocking medications, calcium antagonists for 48 h, and nitrates for 12 h before examination. For patients undergoing a pharmacological stress test, consuming caffeine was not allowed for 24 h before testing. Rest and peak stress heart rate and blood pressure were recorded; monitoring of heart rate, rhythm, and ECG were performed all along the stress protocol. Physical test endpoints were achievement of 85% maximal predicted heart rate, horizontal or down-sloping ST-segment depression > 2 mm, ST-segment elevation > 1 mm, moderate to severe angina, systolic blood pressure decrease > 20 mm Hg, blood pressure > 230/120 mm Hg, dizziness, or clinically important cardiac arrhythmia. At peak exercise or 4 min after dipyridamole infusion, a bolus of ^99m^Tc-sestamibi was intravenously injected. In case of chest pain or other symptoms, as well as significant ST depression, 100 mg of aminophylline was administered intravenously.

### MPI quantification

Total perfusion deficit (TPD), incorporating the extent and severity of perfusion defects and expressed as a percentage of the left ventricular (LV) myocardium, was calculated by an automated software program (e-soft 2.5, QGS/QPS, Cedars-Sinai Medical Center, Los Angeles, CA), using standardized segmentation of 17 myocardial regions; moreover, LV ejection fraction (EF) was automatically computed. The ITPD was defined as stress TPD—rest TPD. Both regional TPD and ITPD were calculated separately for each vascular territory: left anterior descending (LAD), left circumflex (LCx), and right coronary artery (RCA) [17].

### Coronary angiography

The decision to refer patients to coronary angiography after MPI was made by the referring physicians. Coronary angiography was performed within 90 days before MPI in 242 patients and within 90 days after in 348 patients, using the standard Judkins method. No patients studied in the 3 months before MPI received treatment during coronary angiography. Results of coronary angiography were visually interpreted by experienced cardiologists, and significant CAD was considered in the presence of luminal diameter stenosis ≥ 50% in at least one of the three major vascular territories [18, 19].

### Follow-up

Patient follow-up was prospectively obtained by a questionnaire assessed during a phone call to all patients and general practitioners or cardiologists and by review of the hospital or physicians’ records by individuals blinded to the patient’s test results. The outcome was a composite end point of cardiac death, target vessel-related myocardial infarction, or late coronary revascularization (> 6 months after MPI), whichever occurred first. The cause of death was confirmed by a review of the death certificate, hospital chart, or physician’s records. Death was considered of cardiac origin if caused by acute myocardial infarction, congestive heart failure, valvular heart disease, sudden cardiac death, and cardiac interventional/surgical procedure related. Myocardial infarction was defined when more than 2 of the following 3 criteria were met: chest pain or equivalent symptom complex, positive cardiac biomarkers, or typical electrocardiographic changes [20]. To define the event as vessel-related, data of patients with an event at follow-up were blindly reviewed, and the event was unequivocally assigned to the culprit’s vessel in case of acute myocardial infarction and target-vessel revascularization. In case the identification of the culprit’s vessel was not possible or feasible (i.e., cardiac death, no coronary angiography performed, or non-ST segment elevation myocardial infarction in patients with multivessel disease), the event was referred to all the stenotic vessels of those patients [21]. The date of the last examination or consultation was used to determine the length of follow-up.

### Statistical analysis

Continuous data are expressed as mean ± standard deviation and categorical data as a percentage. A Student two-sample *t*-test and chi-square test were used to compare the differences in continuous and categorical variables, as appropriate. A *P* value < 0.05 (two-sided) was considered statistically significant. The receiver operating characteristics (ROC) area under the curve (AUC) was applied to evaluate the diagnostic ability of regional ITPD in identifying the vessel-related event and to establish the best trade-off between sensitivity and specificity for different cutoff points of regional ITPD. The optimal cutoff point was determined by the ROC curve based on the Youden index. Hazard ratios with a 95% confidence interval (CI) were calculated by univariable and multivariable Cox regression analysis. Variables showing a *P* value < 0.05 at univariable analysis were considered for multivariable analysis. Annualized event rate (AER), expressed as % person-years or as % vessel-years, was calculated as the cumulative number of events divided by person-time or vessel-time. Event-free survival curves were obtained by the Kaplan–Meier method and compared with the log-rank test. Statistical analysis was performed using STATA software (ver. 12, College Station, TX).

## Results

Follow-up was 92% complete, leaving 541 subjects for the analysis. During a median follow-up of 27 months (range 7–56 months), 31 cardiac events occurred (6% cumulative event rate with an annual event rate of 2.2% person-years). The events were cardiac deaths in 4 (13%) patients, nonfatal myocardial infarctions in 7 (23%), and late coronary revascularizations in 20 (64%) subjects. Clinical characteristics and imaging findings of patients with and without events are summed in Table 1. Patients with events had similar clinical features and cardiovascular risk factors as compared to patients without events. However, a higher prevalence of significant CAD on coronary angiography was observed in patients with events. In particular, 190 patients had single-vessel disease, 78 patients had 2-vessel disease, and 28 patients had 3-vessel disease. Patients with events also had higher values of summed stress score, summed difference score, stress TPD, and ITPD compared to patients without events.

**Table 1:** Patients’ characteristics and imaging findings according to events

	All patients (*n* = 541)	With events (*n* = 31)	Without events (*n* = 510)	*P* value
Age (years)	64 ± 10	64 ± 9	64 ± 10	0.93
Male gender, *n* (%)	431 (80)	26 (84)	405 (79)	0.65
Diabetes mellitus, *n* (%)	177 (33)	12 (39)	165 (32)	0.55
Hypertension, *n* (%)	503 (93)	29 (93)	474 (93)	1.00
Dyslipidemia, *n* (%)	469 (87)	24 (77)	445 (87)	0.17
Smoking history, *n* (%)	346 (64)	24 (77)	322 (63)	0.05
Family history of CAD, *n* (%)	317 (59)	17 (55)	300 (59)	0.71
Angina, *n* (%)	175 (32)	12 (39)	163 (32)	0.44
Previous infarction, *n* (%)	324 (60)	8 (26)	316 (62)	0.33
Previous revascularization, *n* (%)	380 (70)	10 (32)	370 (73)	< 0.05
CAD ≥ 50%, *n* (%)	296 (55)	28 (90)	268 (53)	< 0.001
1 vessel, *n* (%)	190 (35)	13 (42)	177 (35)	
2 vessel, *n* (%)	78 (14)	10 (32)	68 (13)	
3 vessel, *n* (%)	28 (5)	5 (16)	23 (5)	
Summed stress score	6.15 ± 8.56	10.03 ± 9.82	5.91 ± 8.43	< 0.01
Summed rest score	4.40 ± 7.80	5.81 ± 8.80	4.32 ± 7.73	0.30
Summed difference score	1.64 ± 2.59	3.90 ± 3.87	1.50 ± 2.42	< 0.001
TPD stress (%)	9.06 ± 12.7	13.55 ± 13.4	8.78 ± 12.7	< 0.05
TPD rest (%)	6.61 ±13.2	7.58 ± 12.3	6.55 ± 13.3	0.67
ITPD (%)	2.74 ± 4.35	5.94 ± 5.60	2.54 ± 4.19	< 0.001
Stress LV ejection fraction (%)	54 ± 14	50 ± 13	54 ± 14	0.18
Rest LV ejection fraction (%)	53 ± 14	50 ± 13	53 ± 14	0.23

Values are expressed as mean value ± standard deviation or as the number (percentage) of subjects

*CAD*, coronary artery disease; *TPD*, total perfusion deficit; *ITPD*, ischemic TPD; *LV*, left ventricular

### Per-vessel imaging findings

**Fig. 1:**
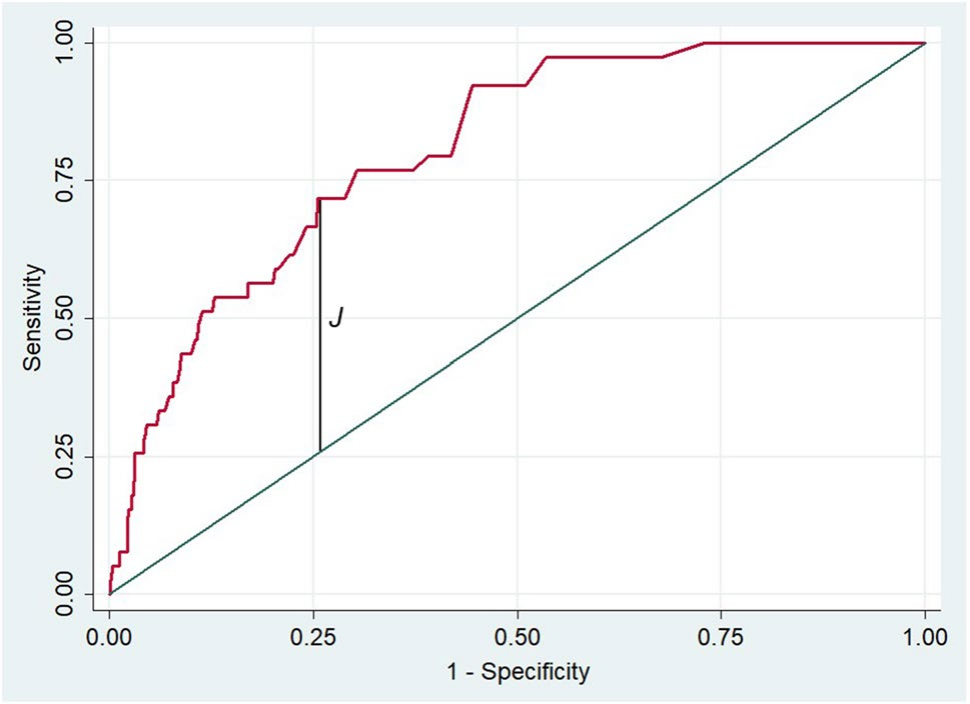
Receiver operating characteristic area under the curve (AUC) for the identification of obstructive CAD using regional ischemic total perfusion deficit (ITPD). A regional value of 2.0% calculated by the Youden index (J) provided the best trade-off for identifying a vessel-related event

Of the overall 1623 vessels analyzed, 430 (27%) showed CAD ≥ 50% on coronary angiography, while 1193 (73%) did not. The vessel-related events were observed in 39 (2%) vessels. In particular, 6 patients experienced events on multiple vessels: 2 patients underwent PCI on 2 vessels during follow-up, and among 4 cardiac deaths, the event was assigned to all the stenotic vessels of each patient (3 vessels for 2 patients and 2 vessels for 2 patients). Regional imaging findings according to vessel-related events are reported in Table 2. The prevalence of CAD ≥ 50%, regional stress TPD, and regional ITPD was significantly higher in vessels with events as compared to those without (both *P* < 0.001). The AUC for regional ITPD for the identification of vessel-related events was 0.81 (95% CI, 0.75–0.86). An ITPD value of 2.0% provided the best trade-offs for identifying vessel-related events (Fig. 1).

**Table 2:** Regional imaging findings according to vessel-related events

	All vessels (*n* = 1623)	With events (*n* = 39)	Without events (*n* = 1584)	*P* value
CAD ≥ 50%, *n* (%)	430 (26)	34 (87)	396 (25)	< 0.001
TPD stress (%)	3.06 ± 5.51	6.33 ± 6.49	2.98 ± 5.46	< 0.001
LAD (%)	5.09 ± 8.06	7.75 ± 8.52	5.01 ± 8.04	0.16
LCx (%)	2.07 ± 3.35	5.75 ± 4.13	1.99 ± 3.30	< 0.001
RCA (%)	2.00 ± 2.88	4.76 ± 3.45	1.95 ± 2.84	< 0.005
ITPD (%)	1.36 ± 1.80	3.42 ± 2.74	1.30 ± 1.74	< 0.001
LAD (%)	1.97 ± 2.33	3.58 ± 2.75	1.92 ± 2.30	< 0.005
LCx (%)	1.01 ± 1.33	3.23 ± 2.18	0.97 ± 1.27	< 0.001
RCA (%)	1.09 ± 1.41	3.53 ± 3.20	1.05 ± 1.31	< 0.001

Values are expressed as mean value **±** standard deviation

*CAD*, coronary artery disease; *TPD*, total perfusion deficit; *ITPD*, ischemic TPD; *LAD*, left anterior descending artery; *LCx*, circumflex artery; *RCA*, right coronary artery

### Predictors of vessel-related outcome events

**Fig. 2:**
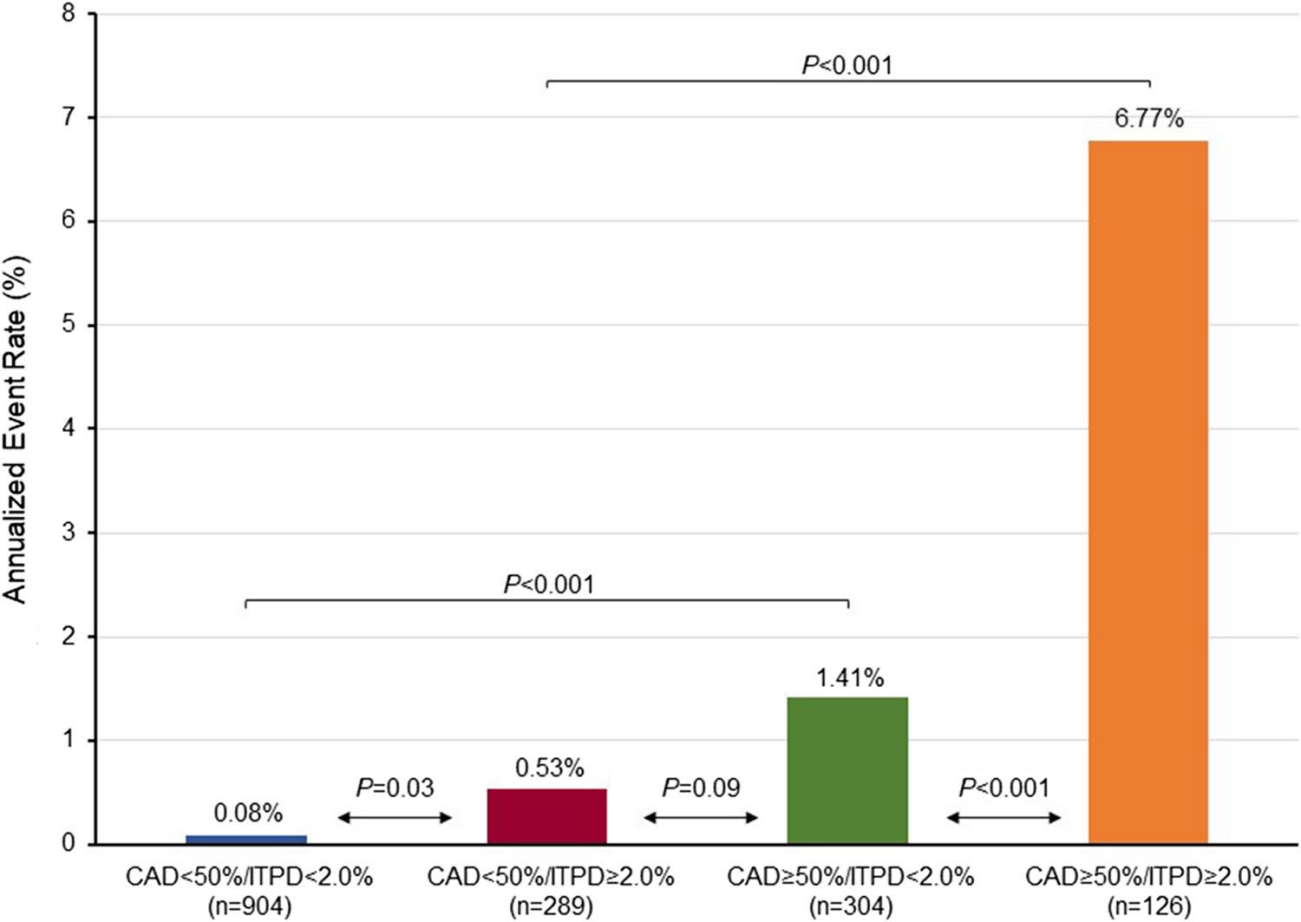
Annualized event rates (AER) in vessels categorized according to the presence of significant CAD and ITPD cutoff

**Fig. 3:**
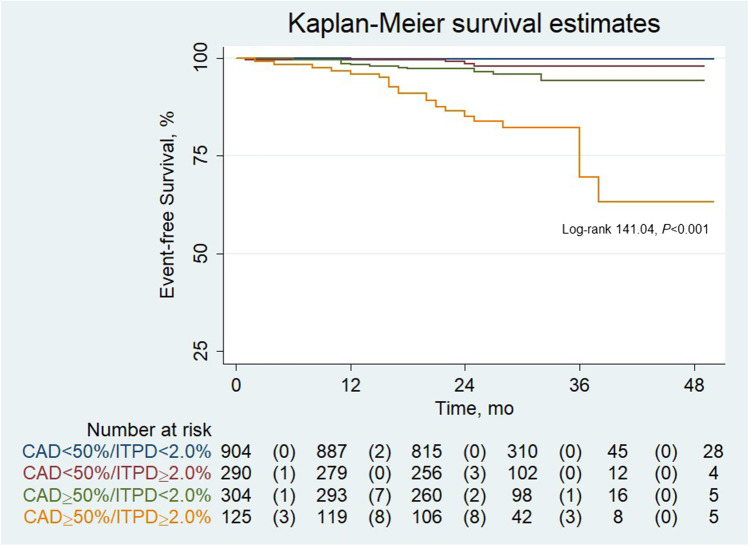
Kaplan–Meier event-free survival curves in vessels categorized according to the presence of significant CAD and ITPD cutoff

The results of Cox regression univariable and multivariable analysis are depicted in Table 3. As shown, CAD ≥ 50%, and ITPD ≥ 2.0% were significant predictors of vessel-related events. At multivariable analysis, both CAD ≥ 50% and ITPD ≥ 2.0% resulted in independent predictors of events. The AER according to CAD and ITPD are reported in Fig. 2. Although the vessels with CAD ≥ 50% and ITPD ≥ 2.0% showed the highest AER, those with CAD < 50% and ITPD ≥ 2.0% showed significantly higher AER as compared to those with ITPD < 2.0% (*P* < 0.05). Finally, event-free survival analysis by the Kaplan–Meier method was performed according to CAD and ITPD as shown in Fig. 3. The worst prognosis was observed in vessels with CAD ≥ 50% and ITPD ≥ 2.0% (log-rank 141.04, *P* < 0.001).

**Table 3:** Univariable and multivariable predictors of vessel-related events

	Univariable analysis		Multivariable analysis	
	Odds ratio (95% CI)	*P* value	Odds ratio (95% CI)	*P* value
CAD ≥ 50%	19.371 (7.575–49.538)	< 0.001	16.610 (6.469–42.643)	< 0.001
ITPD ≥ 2.0%	5.341 (2.776–10.275)	< 0.001	3.512 (1.684–7.321)	< 0.005

*CI*, confidence interval; *CAD*, coronary artery disease; *ITPD*, ischemic total perfusion deficit

## Discussion

To our knowledge, this is the first study analyzing the prognostic value of regional perfusion assessed by the CZT camera in predicting vessel-related events, in patients with suspected or known CAD and angiographic correlation. Our data highlight that regional ITPD is a significant predictor of vessel-related events. The prognostic significance of stress perfusion abnormalities assessed by the CZT camera in the prediction of adverse cardiac events has been extensively addressed. All published data confirmed that the presence of a normal scan is associated with a good outcome, whereas the degree of myocardial perfusion defect is a good predictor of cardiovascular events [10–13]. Engbers et al. [22] in 2017 demonstrated, in a very large cohort of 4057 patients with suspected CAD undergoing MPI with a CZT SPECT camera, that cardiovascular events occurred more in patients with abnormal than in those with normal MPI. Moreover, the events were more frequent along with the increase in the extension of reversible defects, as well as differences in terms of AER, were observed between small and large total perfusion defects. In a recent prospective registry of patients with suspected or known CAD [23], it has been demonstrated that the extent of global stress perfusion abnormalities detected by CZT SPECT had the best accuracy in predicting cardiovascular death and myocardial infarction. In particular, a summed stress score of >8 was a strong predictor of future adverse events, and the presence of ischemia involving > 10% of the myocardium was also associated with all-cause death and late revascularization. Similarly, Lima et al. [11] demonstrated that in 2930 patients with known or suspected CAD, the event rates were higher in patients showing greater extension of perfusion defects and ischemia. Accordingly, in our study population, patients experiencing cardiovascular events during the follow-up had a greater extent and severity of myocardial perfusion abnormalities. In our population, LVEF was not associated with events, data explained by the relatively normal values of LVEF in our study cohort. While previous studies [11, 23] outlined the prognostic value of myocardial perfusion abnormalities assessed by CZT cameras, no studies performed a regional analysis evaluating the potential interplay between the perfusion results and angiographic findings. In the current study, we analyzed the role of the regional TPD automatically provided by the software. Our findings are not greatly different from those reported by Gimelli et al. [23], considering that TPD and ITPD are derived from summed stress and summed difference score, respectively. Our results demonstrate that both regional stress TPD and ITPD were significantly higher in vessels with vessel-related events compared to those without. In particular, we found that an ITPD threshold of 2.0% provided the best trade-off between sensitivity and specificity for identifying vessel-related events, and this threshold resulted in an independent predictor of a vessel-related event at multivariable analysis. A previous report [24] investigated the relationship between the prognostic value of CZT MPI and the presence of obstructive CAD on coronary angiography, demonstrating that patients with abnormal MPI findings and no obstructive CAD showed similar prognosis to patients with obstructive CAD and normal MPI. These per-patient results are consistent with our per-vessel analysis in which vessels with CAD < 50% and ITPD ≥ 2.0% had similar AER as compared to vessels with CAD ≥ 50% and ITPD < 2.0%. However, in our study, the presence of ITPD ≥ 2.0% even in absence of significant CAD was associated with a higher AER and a lower event-free survival as compared to vessels with ITPD < 2.0%. Similar results were found using PET/CT by Zampella et al. [25] in a recent study evaluating the prognostic role of ITPD in predicting lesion-related outcomes in patients with suspected CAD. The combined evaluation of multiple parameters derived from MPI such as regional coronary atherosclerosis and vascular function showed incremental value in predicting the occurrence of lesion-related events in the presence of significant CAD. Accordingly, in our study, it emerged that by combining anatomic information from coronary angiography with MPI findings, a better risk stratification can be provided. Thus, the potential integration of multiple parameters to optimize risk stratification of patients with these noninvasive techniques is desirable to increase the usefulness of regional assessment for prognostic evaluation of patients with suspected or known CAD.

## Conclusion

Regional myocardial perfusion assessed by the CZT camera demonstrated good reliability in predicting vessel-related events in patients with suspected or known CAD. In particular, an ITPD ≥ 2.0% resulted in an independent predictor in identifying high-risk vessels. These findings suggest that using a regional evaluation of myocardial perfusion may help in refining risk stratification and in identifying high-risk coronary stenosis at higher risk of events, in which a change in a patient management could be hypothesized.

## Abbreviations

MPI: Myocardial perfusion imaging
CZT: Cadmium zinc telluride
CAD: Coronary artery disease
SPECT: Single-photon emission computed tomography
ITPD: Ischemic total perfusion deficit
PET/CT: Positron emission tomography/computed tomography
ROC: Receiver operating characteristics
AUC: Area under the curve
CI: Confidence intervals
AER: Annualized event rate
